# The impact of hydrothermal carbonization on the surface functionalities of wet waste materials for water treatment applications

**DOI:** 10.1007/s11356-020-08591-w

**Published:** 2020-04-18

**Authors:** Mirva Niinipuu, Kenneth G. Latham, Jean-François Boily, Magnus Bergknut, Stina Jansson

**Affiliations:** 1 Department of Chemistry, Umeå University, 90187 Umeå, Sweden; 2 Industrial Doctoral School, Umeå University, 90187 Umeå, Sweden; 3 MTC-Miljötekniskt Center AB, Dåva Energiväg 8, 90595 Umeå, Sweden

**Keywords:** Hydrochar, Surface properties, Paper mill sludge, Digested sludge, Horse manure, Adsorption

## Abstract

Hydrothermal carbonization (HTC) is an energy-efficient thermochemical process for converting wet waste products into value added materials for water treatment. Understanding how HTC influences the physicochemical properties of the resultant materials is critical in optimizing the process for water treatment, where surface functionality and surface area play a major role. In this study, we have examined the HTC of four wet waste streams, sewage sludge, biosludge, fiber sludge, and horse manure at three different temperatures (180 °C, 220 °C, and 260 °C). The physicochemical properties of these materials were examined via FTIR, SEM and BET with their adsorption capacity were assessed using methylene blue. The yield of solid material after hydrothermal carbonization (hydrochar) decreased with increasing temperature for all samples, with the largest impact on horse manure and fiber sludge. These materials also lost the highest degree of oxygen, while HTC had minimal impact on biosludge and sewage sludge. The differences here were due to the varying compositions of each waste material, FTIR identified resonances related to cellulose in horse manure and fiber sludge, which were not detected in biosludge and sewage sludge. Adsorption capacities varied between 9.0 and 68 mg g^−1^ with biosludge HTC at 220 °C adsorbing the highest amount. Adsorption also dropped drastically at the highest temperature (260 °C), indicating a correlation between adsorption capacity and HTC conditions. This was attributed to the loss of oxygen functional groups, which can contribute to adsorption. These results suggest that adsorption properties can be tailored both by selection of HTC temperature and feedstock.

**Electronic supplementary material:**

The online version of this article (10.1007/s11356-020-08591-w) contains supplementary material, which is available to authorized users.

## Introduction

Environmentally benign disposal of wet waste materials, such as sewage sludge and manures, is an expanding global problem. Currently, 50 million tons of sewage sludge and 1.4 billion tons of manure is produced annually in the EU (Foged et al. 2012; Kelessidis and Stasinakis 2012). In China, 3.8 billion tons of manure is produced annually and sewage sludge production expected to rise to 52.92 million tons by 2020 (Gao et al. 2019). These amounts are expected to further increase with global population and living standards over the next century.

Traditionally, these wet waste materials are disposed in landfill, incinerated, or utilized as fertilizers; however, this is problematic because of contaminates (e.g., heavy metals, ash, pharmaceuticals, personal care products (PPCPs), and pathogens), bans on landfilling combustible materials, and eutrophication/acidification from nitrogen and phosphorus leeching. The high moisture content, up to 97% *w*/*v* (Tasca et al. 2019), also makes transportation costly and limits the disposal of these materials to facilities in proximity to the waste source.

Biological and thermochemical methods, such as anaerobic digestion, torrefaction, slow pyrolysis, pelletization, gasification, liquefaction, and hydrothermal carbonization have been examined to counteract the abovementioned issues. Each method has its own advantages and drawbacks, with hydrothermal carbonization (HTC) being one of the most promising and energy-efficient methods for processing wet waste materials. This is because the thermochemical conversion process in HTC occurs under water, negating the large energy cost of water removal. During HTC, the wet waste materials are heated to temperatures up to the critical point of water (374 °C) in a high-pressure reactor for 2–20 h, although temperatures between 180 and 280 °C are more commonly used. Pressure generated inside the reactor is between 20 and 80 bar and is proportional to the vapor pressure of water and material being carbonized. The result is a solid carbonaceous material, an organic rich water phase and small amounts of gas (CO_2_, CH_4_). The physicochemical properties of the final products depend on the reaction conditions, precursor, and pH, allowing a high level of tuneability (Latham et al. 2014; Tasca et al. 2019; Zhou et al. 2019). Additionally, HTC treatment sterilizes the waste materials, removes water soluble species, stabilizes heavy metals (Fan et al. 2017; Tasca et al. 2019), allows the recovery of phosphorus and nitrogen (Becker et al. 2019), and breaks down pharmaceuticals (vom Eyser et al. 2015, 2016). Overall, HTC is considered a closed cycle approach that can recycle wet waste materials and recover nutrients.

There is a wide range of studies examining the HTC of sewage sludge (Tasca et al. 2019), biogas digestate (Rodriguez Correa et al. 2017), paper mill sludge (Mäkelä et al. 2015; Mäkelä and Yoshikawa 2016), human excrement (Fakkaew et al. 2018), chicken litter, and swine manure (Ro et al. 2018; Zhou et al. 2019) for energy applications (Fakkaew et al. 2018; Ro et al. 2018; Zhou et al. 2019) and soil amendments (Melo et al. 2018). In general, HTC reduces the water content of these solid materials, while concentrating insoluble inorganic species (ash) (Gasco et al. 2018; Tasca et al. 2019; Zhou et al. 2019). Carbon content in the final solid product is dependent on the precursor and not the reaction conditions (Zhou et al. 2019). This is different from lignocellulosic biomass, in which carbon content almost always increases after HTC with increasing temperature (Wilk et al. 2019). HTC treatment increased the highest heating value (Tasca et al. 2019; Zhou et al. 2019); however, ash concentration could be problematic, leading to slagging and fouling issues upon combustion. Despite the wide range of studies on the HTC of wet waste materials, studies examining these materials for higher value applications, such as water treatment, along with in-depth surface studies, are lacking.

Reasons why this is lacking is likely due to the sheer complexity of the surface of wet waste materials. The HTC reaction occurs through water breaking the carbon-oxygen, and to a lesser extent, carbon-carbon bonds between the organic structures present in the waste materials. The degree that this occurs depends on the type of organic structure (e.g., cellulose, lignin, proteins), its physical location (surface or bulk), and the reaction conditions. For insoluble materials, the access to water also needs to be considered, resulting in two pathways. The first is the solubilization of surface organic species into the water, which can be followed by repolymerization onto the surface. The second is the impact of temperature on the bulk structure that does not have access to water. As a result, the surface of the material comprises of a mixture of repolymerized, freshly reacted, and un-reacted regions (Dinjus et al. 2011). It is important to understand the properties of the surface as the adsorption of contaminates to carbon materials is governed through various interactions with surface functional groups, such as hydrogen bonding, dipole-dipole, dipole-induced dipole, and complexation (Fang et al. 2014; Lu et al. 2012; Sun et al. 2012). Stabilized inorganic species contained in the waste materials may also promote anion adsorption and ion exchange (Yang et al. 2014; Yao et al. 2011), leading to increased adsorption capacity.

In this study, we examine how altering the HTC processing conditions impacts the surface functionality of four low-cost waste materials. Two of these have been studied in the literature for their energy potential, sewage sludge and biosludge, and two materials that have rarely been studied, horse manure, and fiber sludge. FTIR, XPS, SEM, and BET have been used to extensively study the surface properties of these materials and the impact of HTC at several temperatures. Additionally, to assess the adsorption potential of these materials, adsorption isotherms with methylene blue were conducted to allow easy comparisons with the literature.

## Materials and methods

### Preparation of hydrochars

Hydrothermal carbon was produced from four feedstocks, fiber sludge, biosludge, sewage sludge, and horse manure. The fiber sludge and biosludge samples were collected from a Swedish paper mill producing bleached pulp, kraftliner, and folding boxboard. Wastewater from pulp and boxboard production lines is sedimented, producing fiber sludge that consists of bleached and unbleached fibers. Water from pre-sedimenting is mixed with low-fiber wastewater and aerated after chlorate reduction and pH adjustment. Sludge sedimented after this process is referred to as biosludge. Anaerobically digested sludge was sampled form a municipal wastewater treatment plant (Umeå, Sweden). A schematic overview of the water treatment process in this water treatment plant was described previously by Östman et al. (2018). Horse manure was collected in a riding school in Vännäs, Sweden. The horse manure intermixed with sawdust, wood chips, hay, and sand from the stables. The elemental composition of these materials is presented in Table S1, Supporting Information. Each material was homogenized and then mixed separately with water until covered to form a slurry. This produced four different slurries with a dry material content between 10 to 22% (Table S2, Supporting Information).

Hydrothermal carbonization was carried out by adding 600 mL of slurry to a 1-L non-stirred stainless-steel HTC reactor (Amar Equipments Pvt. Ltd.) and heated to 180, 220, or 260 °C for 2 h. After 2 h, the reactor was cooled by blowing pressurized air over the reactor. The solid product was filtered and then washed by adding them to clean deionized water and agitated for 1 h (~ 100 g char L^−1^). The washed solutions were again filtered, rinsed with 200 mL of deionized water, and finally dried at 105 °C overnight in an oven. This process resulted in twelve chars from the four different precursors at three temperatures.

The pH of the materials was measured (PHM290 pH-meter, Meter lab) after measuring 0.5 g of each hydrochar shaken in 10 mL deionized water for 1.5 h (performed in triplicate), as described by Rajkovich et al. (2011).

### Physical characterization

Surface morphology was examined via scanning electron microscopy (SEM, Carl Zeiss Evo) operating in low vacuum mode. Images were taken with the fitted variable pressure secondary electron detector.

Brunauer−Emmett−Teller (BET) surface area was determined with a Micromeritics TriStar 3000 gas adsorption analyzer after degassing 50–100 mg of each dry sample. Degassing was performed for 2 h at 120 °C, under a continuous nitrogen flow, using a Micromeritics Smart Prep degassing unit. The specific surface areas were obtained by applying the BET to multi-point N_2_(g) adsorption/desorption isotherms.

### Chemical characterization

Fourier transform infrared (FTIR) spectra were collected using a Vertex 70v (Bruker Co., Germany) spectrometer equipped with an attenuated total reflectance (ATR) cell (single bounce diamond; Golden Gate, Specab). Dried and homogenized samples were pressed onto the ATR cell using a sapphire anvil. Absorption spectra were obtained (wavenumbers 600–4000 cm^−1^, resolution 4 cm^−1^) in 100 co-added scans recorded under vacuum at room temperature.

The surface composition of the chars was determined via X-ray photoelectron spectroscopy (XPS) equipped with a monochromatic Al Kα source operated at 120 W. The spectra were collected with a Kratos Axis Ultra DLD electron spectrometer and processed with Kratos software. An analyzer pass energy of 160 eV and a pass energy of 20 eV were used for acquiring survey spectra and individual photoelectron lines, respectively. A spectrometer charge neutralization system was used to stabilize the surface potential. The binding energy (BE) scale was referenced to the C 1s line of aliphatic carbon, which was set to 285.0 eV. Using a Ni spatula, powder samples for the analysis were gently pressed into a pellet affixed to a sample holder. The limit of detection (LOD) was ~ 0.1 at. %.

### Methylene blue adsorption

Adsorption isotherm tests were carried out for all materials carbonized at 220 °C and for horse manure and biosludge carbonized at 180 °C and 260 °C. Methylene blue stock solution was prepared in ultrapure water, c = 1000 mg L^−1^, which was further diluted to the following concentrations: 200, 160, 120, 80, 60, 40, and 10 mg L^−1^. Additionally, tests in ultrapure water were carried out. Adsorption tests were started by weighing 0.025 g of adsorbent in a polypropylene tube, and 10 mL of methylene blue solution was added. All the samples were shaken in an end-to-end shaker (40 rpm) for 24 h to reach the equilibrium. After that time, the samples were filtered through 0.45 μm filter. The absorbance at 668.0 nm for each sample was measured with Spectronic Unicam Helios Gamma (Thermo Fisher Scientific Inc.) spectrophotometer after diluting (if needed) the samples to the concentration range 1–5 mg L^−1^.

The data was further investigated by fitting Freundlich and Langmuir isotherms. The nonlinear form of Freundlich isotherm is expressed as (Freundlich 1907):

$$ {q}_e={K}_f{C}_e^{\raisebox{1ex}{$1$}\!\left/ \!\raisebox{-1ex}{$n$}\right.} $$

where *q*_*e*_ is the amount (mg g^−1^) of methylene blue at equilibrium, *K*_*f*_ is the Freundlich constant (mg g^−1^)(L/mg)^−1/*n*^) (indicating adsorption capacity), *C*_*e*_ is methylene blue concentration in the solution at the equilibrium (mg L^−1^), and *n* represents the nonlinearity of the fitting.

Langmuir equation nonlinear form is expressed as follows (Langmuir 1918):

$$ {q}_e=\frac{Q_{max}{K}_l{C}_e}{1+{K}_L{C}_e} $$

where *Q*_max_ is the maximum adsorption capacity (mg g^−1^) and *K*_*L*_ is the Langmuir constant.

## Results and discussion

### Yield of solid material after HTC

**Fig. 1:**
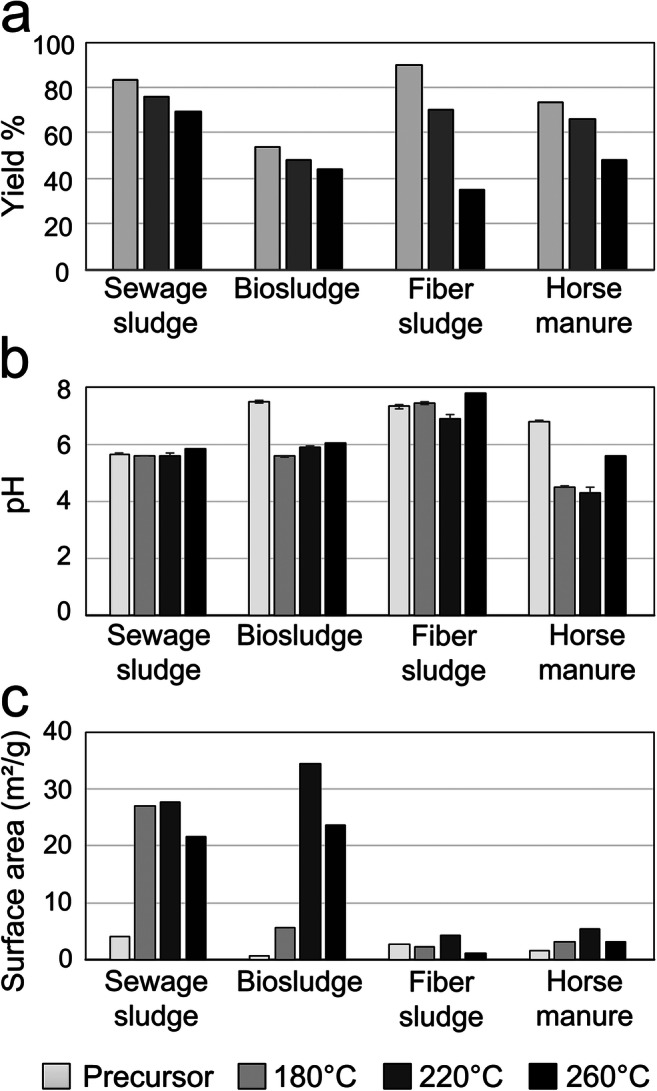
**a** Yield, **b** pH (averages, with error bars corresponding to ± 1 st. dev.), and **c** surface area of each char. The yield and surface area are based on a single measurement, whereas the pH was obtained from triplicate measurements

The yield, based on dry weight, varied between 35 and 90%, depending on the temperature and precursor (Fig. 1a). The highest yields were observed for the lowest temperature, an expected result as higher temperatures in HTC typically lead to lower yields (Rodriguez Correa et al. 2017; Yahav Spitzer et al. 2018; Zhou et al. 2019). Differences in yield between the studied materials are related to their composition, as each material contains a varying mixture of lignocellulosic and inorganic components (elemental composition shown in Table S1). These differences were determined using known FTIR resonances for cellulose and lignin with the inorganic fraction determined using XPS (“Characterization of the surface functionalities” section). Fiber sludge and horse manure are rich in lignocellulosic materials (Dong et al. 2016; Muhammad Nasir and Mohd Ghazi 2015), whose components (hemicellulose, cellulose, and lignin) decompose at different temperatures (Atta-Obeng et al. 2017; Bobleter 1994; Kang et al. 2012). The decomposition of each of these components is also accelerated at higher temperatures and can be seen here from the increase in loss between temperature steps (180 to 220 °C is 20% and 220 to 260 °C is 35% for fiber sludge).

The impact of increasing the temperature is not as pronounced in the bio- and sewage sludge samples; however, the initial loss at 180 °C is greater. Again, this is due to composition as these materials are known to contain a mixture of cellulose, hemicellulose lignin, proteins, carbohydrates, lipids, and inorganics (Mäkelä and Yoshikawa 2016; Rodriguez Correa et al. 2017; Tasca et al. 2019). Proteins, lipids, and carbohydrates have been found to make up a 33.4, 6.6 and 3.3% of sewage sludge, respectively, although the exact amount will be variable between sludges (Inoue et al. 1996). Apart from cellulose, lignin, and the inorganic fraction, the remaining components decompose under HTC at 180 °C. This results in a higher fraction of organic species dissolved in the water at lower temperatures, reducing the yield compared to horse manure and fiber sludge. Losing these components at the lowest temperature also explains the limited impact of temperature increases, as the main reactants have already been removed.

### Surface area and morphology

**Fig. 2:**
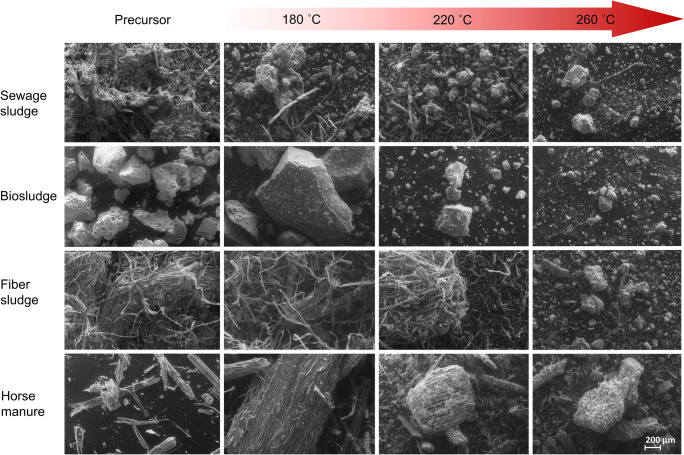
SEM images (magnification × 150) of the studied materials

SEM images revealed that each of the precursors had different morphologies (Fig. 2). For the digested precursors, the sewage sludge displayed fibers intermixed with amorphous particles, while the biosludge only displayed large amorphous particles. Fibers were the only thing present in fiber sludge, while the horse manure resembled lignocellulosic biomass in appearance. After HTC, the size of the particles decreased with increasing temperature, which was especially noticeable in sewage sludge, where the amorphous particles decomposed leaving the fibrous sections intact at ≤ 220 °C. Fiber sludge also underwent a large change at 260 °C with almost all the fibrous material being decomposed, correlating with the large yield decrease (35%). Horse manure did not display the same level of change, with the distinctive plant macrocellular structure still being apparent at 260 °C. This is comparable to other studies examining the HTC of lignocellulosic materials and is attributed the difficulty in breaking down lignin at lower temperatures under HTC conditions (Kambo and Dutta 2015; Kang et al. 2012).

The specific surface area measurements revealed that the porosity of the digested sludge chars is higher than that of fiber sludge and horse manure (Fig. 1c), with the surface area increasing during HTC treatment. For all feedstocks, the highest surface areas were found for chars generated at 220 °C, with the decrease at 260 °C likely due to pore collapse and the condensing aromatic structure (Fang et al. 2014).

### Characterization of the surface functionalities

Previous studies have shown that sludge contains a mixture of proteins, lipids, lignin, carbohydrates, and inorganic components (Alvarez et al. 2015; Inoue et al. 1996; Xia et al. 2018), while manures consist of partially digested lignocellulose, nitrogen, phosphorus, and alkali metals (Muhammad Nasir and Mohd Ghazi 2015; Shen et al. 2015). Thus, the chemical composition of the precursors is likely to contain a mixture of organic, inorganic, and lignocellulosic components. Some of these components are relatively stable under HTC conditions (e.g., Al/Si clays), while others will react and change (e.g., proteins, hemicellulose, lipids, hydroxyls, alkyls, ethers, amines). To examine this, FTIR and XPS were used to understand the surface composition of the precursors, and the produced HTC chars.

**Fig. 3:**
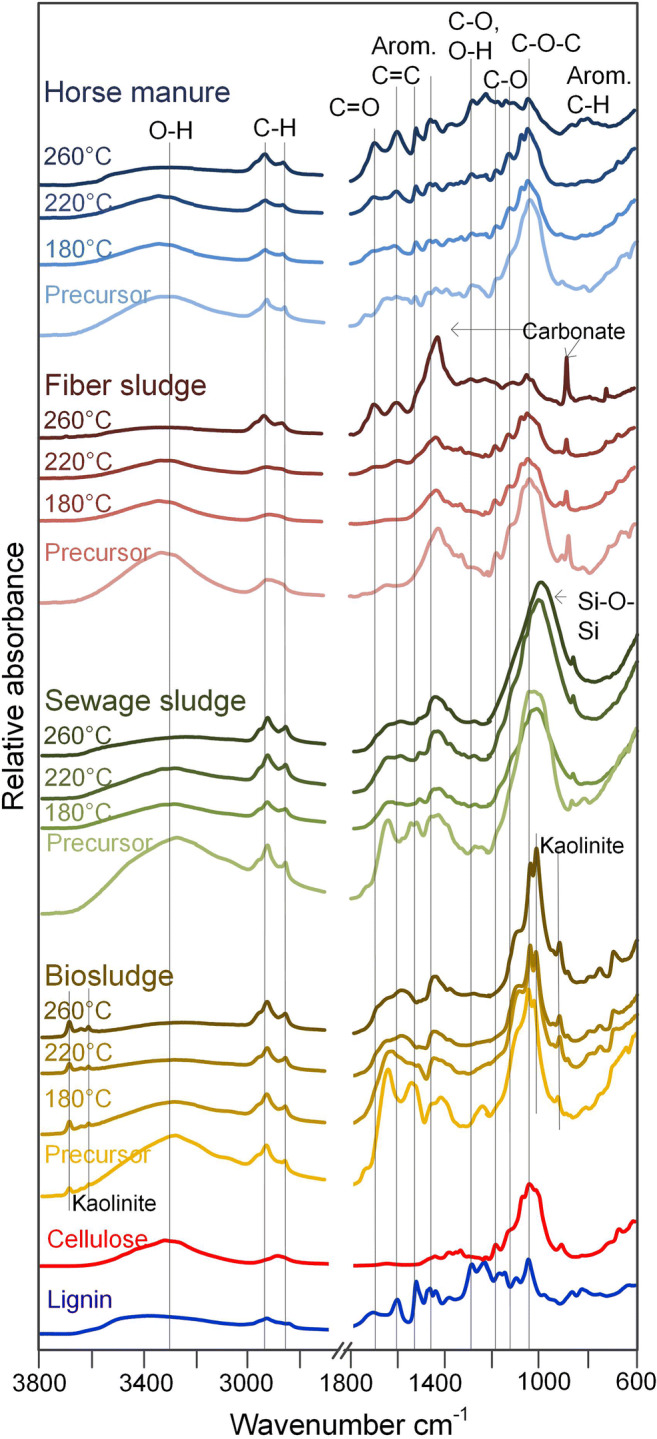
ATR-FTIR spectra for the temperature series, including feedstocks of horse manure, fiber sludge from a paper mill, sewage sludge, and digested sludge from a paper mill. Cellulose and lignin spectra are shown for comparison

Each of the precursors had a unique FTIR spectra (Fig. 3) with few similarities. These were the O-H stretching band at ~ 3300 cm^−1^, CH_3_/CH_2_ stretching band at 2920 cm^−1^ and 2850 cm^−1^ (Chen and Chen 2009), and a large band comprised of several peaks at ~ 1030 cm^−1^. This large band is associated with C-O- linkages in cellulose and to a lesser extent lignin, but also where Si-O-Si resonances are located. For comparison, the FTIR spectra of cellulose and lignin are also displayed in Fig. 3. The most important resonances here for determining the presence of lignin are at 1506 cm^−1^ for C=C aromatic and between 1350 and 1100 cm^−1^ for different C-O ring configurations (1327, 1267, 1219, and 1123 cm^−1^). For cellulose, O-H resonances at 3290 and 3350 cm^−1^ are distinct from lignin, as well as 1155 cm^−1^ for C-O-C and 892 cm^−1^ for C-H bonds (Méndez et al. 2009). Cellulose also has resonances at 1110, 1160, 1055, and 1026 cm^−1^ corresponding to C-O-C, C-O, and O-H (Méndez et al. 2009); however, these are not easily observable in all samples due to the inorganic content. Every precursor has a O-H band at 3350 or 3290 cm^−1^, and all but sewage sludge have a peaks at 1155 cm^−1^ and 892 cm^−1^, indicating the presence of cellulose. Other than fiber sludge, each precursor has a resonance at 1506 cm^−1^ and bands in the 1350–1100 cm^−1^ region related to lignin. This suggests that fiber sludge predominately contains cellulosic materials, while the other precursors contain a mixture of lignin and cellulose.

Regarding the inorganic fraction, fiber sludge contains carbonate (1416 cm^−1^ and 872 cm^−1^ (Smidt and Parravicini 2009)), while biosludge contains kaolinite (3620–3695, 1030, 1005, and 912 cm^−1^ OH stretching, Si-O-Si in-plane vibrations and OH bending, respectively) (Cheng et al. 2015). Both compounds are commonly used additives in the pulping industry (Mäkelä et al. 2015). Sewage sludge also contains bands related to aluminosilicates at ~ 1000 cm^−1^; however, the lack of sharp bands in this region did not allow for the specific aluminosilicate to be identified.

After HTC, the largest intensity changes occurred from the loss of oxygen-containing functional groups (Fig. 3, Table S3, Supporting information) coupled with an increase in vibrations associated with aromatic functionalities. At 180 °C, only very minor changes in the fiber sludge and horse manure are observed, while sewage and biosludge lose the peak at 1637 cm^−1^ associated with C=O on amides in proteins and lipids (Su et al. 2015). The decomposition of organic components is the reason for the large yield loss at 180 °C for the sludges (Alvarez et al. 2015; Inoue et al. 1996; Xia et al. 2018).

At 220 °C, vibrations associated with aromatic functionalities are also observed to increase at 220 °C in the other materials, suggesting carbonization, or the repolymerization of organic species from the water onto the surface has started to occur at this temperature. Despite the higher temperature, characteristic peaks for cellulose (1110, 1160, 1055, and 1026 cm^−1^ corresponding to C-O-C, C-O, and O-H) (Méndez et al. 2009) are still observable in fiber sludge. Additionally, the band at ~ 1030/1008 cm^−1^ for C-O-C and C-OH functionalities appears to have not been affected at 220 °C in any of the materials. This suggests that despite the temperature being high enough for cellulose degradation (Bobleter 1994), the 2-h residence time may not have been long enough to complete the decomposition.

At 260 °C, the peaks associated with cellulose and C-O-C/C-OH at ~ 1030/1008 cm^−1^ are substantially diminished in the horse manure and fiber sludge but remain unaffected in the digested sludges. This is due to the aluminosilicates in the digested sludges that also display peaks in this region, specifically Si-O stretching (1030 and 1008 cm^−1^) and Al-OH (939 and 912 cm^−1^).

**Fig. 4:**
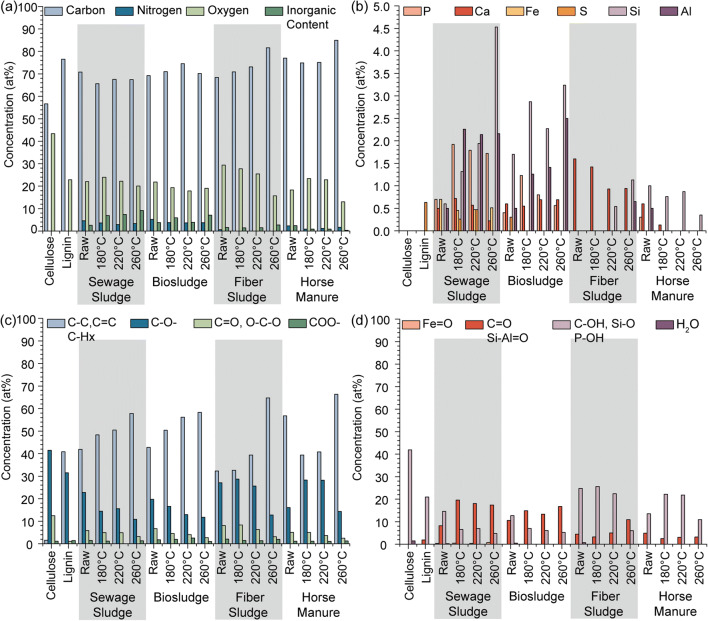
XPS analysis of lignin, cellulose, sewage sludge, biosludge, fiber sludge, horse manure and HTC chars at 180, 220, and 260 °C. Concentration of **a** C, O, N, and other detected elements; **b** inorganic elements; **c** functionalities under C1s peak; and **d** functionalities under O1s peak

To confirm this, XPS was utilized to quantitatively examine the inorganic compounds on the surface of these materials (Fig. 4b). The highest concentration was found in the sewage and biosludge materials, with phosphorus, calcium, iron, sulfur, silicon, and aluminum detected (Fig. 4b). Fiber sludge displayed calcium, silicon, and aluminum and horse manure had a mixture of phosphorous, calcium, silicon, and aluminum. Using the peak position of each of these elements, the dominant form was identified as PO_4_ (133 eV), CaCO_3_ (347.2 eV), Fe_2_O_3_ (710.8 eV), R-SH/SO_4_ (164/166.5), and Al_2_Si_2_O_5_ (102.7 eV, kaolinite). HTC treatment concentrated several of these compounds, while others were removed from the final material. Aluminum and silicon were concentrated with increasing temperature in all samples except for horse manure, while calcium was progressively removed. This is likely to be due to the solubility of each of these compounds, where CaCO_3_ can move into the water solution as kaolinite is hydrophobic and will concentrate in the char. Interestingly, phosphorus is initially concentrated at 180 °C before being systematically removed as the temperature increases. Orthophosphate and long-chain polyphosphates have been determined to be the prevailing forms of phosphate in sludge, while inorganic orthophosphates are dominant in the material after HTC (Huang and Tang 2015). This is also observed here as the phosphorus peak sits at 133.0 ± 2 eV for each sample, representative of for orthophosphates (PO_4_^−3^). Considering the degree that phosphorus increases initially like aluminosilicate (i.e., the concentrations of Si and Al), it is possible that phosphorous is bound into the aluminosilicates or iron and is slowly removed with temperature. A similar finding was observed by Huang and Tang using P K-edge XANES at different residence times (Huang and Tang 2016). The HTC of biosludge (activated sludge) at 225 °C increased the Fe-associated P from 2.3% in the raw sludge to 13% and 4.4% at 4 h and 16 h residence time, respectively. AlPO_4_ groups were observed to be stable with increased residence time, but HTC did concentrate the degree of these groups. The phosphorus peak in this study also broadened slightly with increasing temperature, suggesting other phosphate group formation. This was also observed by Huang and Tang (2015), where HTC increased the amount of each phosphorus type.

To examine the organic fraction, the raw materials were initially compared to the XPS of cellulose and lignin. Each of the raw samples had an oxygen concentration lower than cellulose, suggesting that none of the raw samples consisted of purely cellulose. This is further confirmed when looking at the C1s and O1s (Fig. 4c and d and Table S4, Supporting Information), where cellulose is shown to consist mainly of C-O- linkages, while all other raw samples did not contain the same degree of C-O- linkages. The raw sewage and biosludge samples contain lignin-like oxygens. It should however be noted that the organic oxygen content is lower than inorganic oxygen (e.g., P-O, Si-O, Al-O).

XPS C1s analysis gave a clear insight into the impact of HTC on the organic fraction, as oxygen, and to a lesser extent nitrogen, directly connected to carbon (Fig. 4c). The standard HTC trend is observed here with C-C/C=C/C-H increasing with treatment temperature at the expense of oxygenated functionalities. Sewage and biosludge displayed a steady stepwise increase from the raw materials, whereas there was minimal change in fiber sludge until 260 °C. This is different from what is observed in the literature, where carbon content does not always show a clear correlation with HTC temperature (Wilk et al. 2019; Zhou et al. 2019). These studies do not examine the surface and calculate carbon from the elemental analysis of the bulk, suggesting that the surface potentially becomes increasingly carbonized from repolymerization, while the bulk loses carbon overall. The increase in C-C/C=C/C-H also correlates with the FTIR that displayed an increase in aromatic functionalities at 260 °C with the loss of C-O- groups. The horse manure displayed an entirely different pattern, where the materials produced at 180 and 220 °C have significantly less C-C/C=C/C-H and an increase in C-O- compared to raw horse manure.

Overall, the degree of change in the studied materials was largely dependent on waster material composition. For instance, the digested sludge materials yield differed between 10 and 13% from 180 to 260 °C, while horse manure and fiber sludge lost 26 and 55% in the same temperature range, respectively. The differences in yield were attributed to the composition of each of the materials identified by FTIR and XPS. Each of these materials also contained several inorganic species that were identified by XPS to be PO_4_ (133 eV), CaCO_3_ (347.2 eV), Fe_2_O_3_ (710.8 eV), R-SH/SO_4_ (164/166.5), and Al_2_Si_2_O_5_ (102.7 eV, aluminosilicate). The aluminosilicates were concentrated with increasing temperature, while phosphorus, calcium, and sulfur were progressively removed with temperature.

### Adsorption of methylene blue

Methylene blue adsorption has been, and still is, a commonly used standard test method for activated carbon materials for investigating the surface area and/or the adsorption capacity of the material (Raposo et al. 2009; Standardization 2017). Methylene blue adsorption data is highly abundant, which allows accurate comparisons between different studies. Adsorption studies using environmental pollutants are harder to compare due to the virtually unlimited amount of potential target compounds. Even if methylene blue adsorption might not represent all environmental pollutants, it can provide indications of the affinities of molecules with similar properties (that assumedly have similar interactions with the surface).

**Fig. 5:**
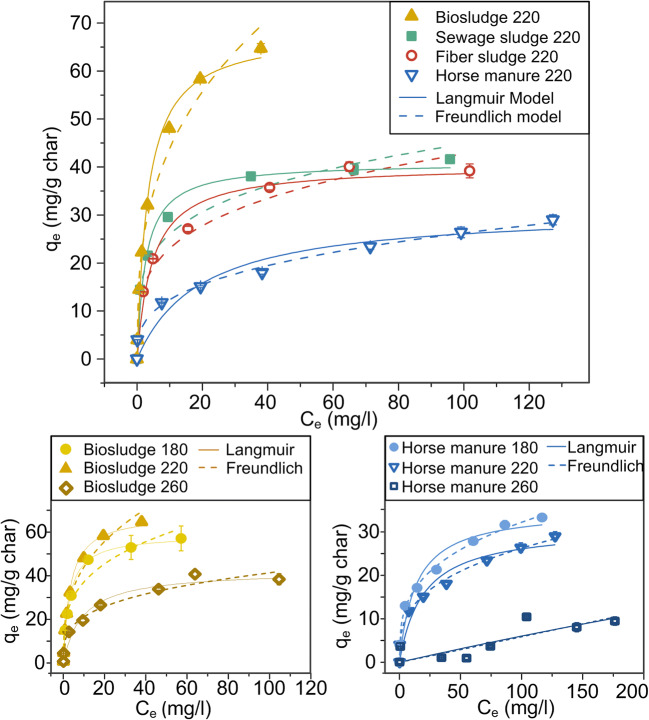
Methylene blue adsorption isotherms for all feedstock materials carbonized at 220 °C (top) and for biosludge (left bottom) and horse manure (right bottom) carbonized at 180 °C, 220 °C and 260 °C, respectively

The adsorption of methylene blue varied substantially between the HTC materials (Fig. 5, Table S5, Supporting Information). Biosludge had the highest adsorption capacity, followed by sewage sludge, fiber sludge, and lastly horse manure.

To examine the impact of temperature, horse manure and biosludge at 180, 220, and 260 °C were directly compared (Fig. 5). The adsorption for both materials was similar at 180 and 220 °C, with a sharp decrease at 260 °C. This decrease can be explained through the loss of two important adsorption properties at 260 °C, surface area and functional groups. The importance of surface groups can be easily observed in the biosludge sample, where the surface is 5.6 m^2^ g^−1^ at 180 °C and adsorption capacity is 59 g g^−1^. At 260 °C, the surface increases to 24 m^2^ g^−1^, but the adsorption drops to 42 g g^−1^. At 260 °C, the level of carbon-oxygen surface functionalities decreases by ~ 8 at%. This supports findings from previous studies that suggest that electrostatic interaction between surface functionalities and methylene blue is an important adsorption mechanism on bio/hydrochars (Fan et al. 2017). The decrease at higher temperature also makes it highly unlikely that kaolinite is participating in the adsorption mechanism, as kaolinites maximum concentration is at 260 °C.

Higher pH favors methylene blue removal because of the increasing negative surface charge that attracts positively charged compounds (Rafatullah et al. 2010). The water pH (Fig. 1b), measured by introducing 0.5 g of char to 10 mL of water, varied among chars produced at different temperatures. For digested sludge types, the pH value changed only slightly with the treatment temperature. However, for the horse manure and fiber sludge, the pH was lowest at 220 °C and highest at 260 °C. This concurs with a previous study where the pH of the raw material, poultry litter, was higher compared to the produced hydrochars and the lowest pH was found at carbonization temperatures at 175 °C or 200 °C (within temperature range 150–300 °C) (Ghanim et al. 2016). The properties of feedstock material (i.e., surface functional groups and inorganic species) have an impact on the char pH. This is clearly seen for fiber sludge that has clearly higher pH compared to the other materials, which is likely because fiber sludge contains carbonates. The poor performance of the horse manure chars may be related to the pH (pH = 4.4–5.6), as its pH is lower at 220 °C, despite having a similar degree of surface functionalities and surface area as fiber sludge (pH = 7.0–7.9).

Results from this study were in line with previous studies made with similar materials, such as pyrolyzed sludge (24 mg g^−1^), granular sludge ZnCl_2_ activated biochar (91 mg g^−1^), biochar/AlOOH nanocomposite (85 mg g^−1^), coffee husk hydrochar (35 mg g^−1^), and composted biowaste pyrochar (15 mg g^−1^) (Anceschi et al. 2018; Fan et al. 2017; Ronix et al. 2017; Shi et al. 2014; Zhang and Gao 2013) while the removal capacities for commercial activated carbons may vary from a few hundred milligrams to nearly a thousand milligrams per gram of carbon (Rafatullah et al. 2010).

## Conclusion

Four wet waste materials were studied under different HTC temperatures to examine the impact on surface functionality and methylene blue adsorption. The yield of char was strongly dependent on temperature, with increasing temperature decreasing the yield in all materials. Compared to the anaerobically digested sludge materials, horse manure and fiber sludge underwent more substantial temperature-induced changes in their physicochemical properties. This was attributed to the differences in composition. Fiber sludge and horse manure contained a range of lignocellulosic materials that were easily degraded under HTC. Sewage and biosludge had higher levels of insoluble inorganic components that did not degrade under HTC. The HTC materials had low surface areas, placing a greater role on surface functionalities to provide adsorption sites for methylene blue. The importance of surface functional groups was demonstrated with the biosludge sample, where temperature induced surface area increases from 5.6 (180 °C) to 24 m^2^ g^−1^ (260 °C) actually decrease the adsorption capacity (59 g g^−1^ at 180 °C vs 42 g g^−1^ a 260 °C) due to a loss in oxygen content. Overall, this indicates that understanding how the surface properties of these wet waste materials change with HTC is vitally important in optimizing processes for valorizing these materials in water treatment applications.

## Electronic supplementary material

ESM 1 (PDF 406 kb).

## References

[CR1] Alvarez J, Amutio M, Lopez G, Bilbao J, Olazar M (2015). Fast co-pyrolysis of sewage sludge and lignocellulosic biomass in a conical spouted bed reactor. Fuel.

[CR2] Anceschi A (2018). Sustainable N-containing biochars obtained at low temperatures as sorbing materials for environmental application: municipal biowaste-derived substances and nanosponges case studies. J Anal Appl Pyrolysis.

[CR3] Atta-Obeng E, Dawson-Andoh B, Seehra MS, Geddam U, Poston J, Leisen J (2017). Physico-chemical characterization of carbons produced from technical lignin by sub-critical hydrothermal carbonization. Biomass Bioenergy.

[CR4] Becker GC, Wust D, Kohler H, Lautenbach A, Kruse A (2019). Novel approach of phosphate-reclamation as struvite from sewage sludge by utilising hydrothermal carbonization. J Environ Manag.

[CR5] Bobleter O (1994). Hydrothermal degradation of polymers derived from plants. Prog Polym Sci.

[CR6] Chen B, Chen Z (2009). Sorption of naphthalene and 1-naphthol by biochars of orange peels with different pyrolytic temperatures. Chemosphere.

[CR7] Cheng H, Hou X, Liu Q, Li X, Frost RL (2015). New insights into the molecular structure of kaolinite–methanol intercalation complexes. Appl Clay Sci.

[CR8] Dinjus E, Kruse A, Tröger N (2011). Hydrothermal carbonization - 1. Influence of lignin in lignocelluloses. Chem Eng Technol.

[CR9] Dong Y, Schneider L, Hu T, Jaakkola M, Holm J, Leveque JM, Lassi U (2016). Direct acid-catalysed mechanical depolymerisation of fibre sludge to reducing sugars using planetary milling. Biomass Bioenergy.

[CR10] Fakkaew K, Koottatep T, Polprasert C (2018). Faecal sludge treatment and utilization by hydrothermal carbonization. J Environ Manag.

[CR11] Fan S, Wang Y, Wang Z, Tang J, Tang J, Li X (2017). Removal of methylene blue from aqueous solution by sewage sludge-derived biochar: adsorption kinetics, equilibrium, thermodynamics and mechanism. J Environ Chem Eng.

[CR12] Fang Q, Chen B, Lin Y, Guan Y (2014). Aromatic and hydrophobic surfaces of wood-derived biochar enhance perchlorate adsorption via hydrogen bonding to oxygen-containing organic groups. Environ Sci Technol.

[CR13] Foged H, Flotats Ripoll X, Bonmatí Blasi A, Palatsi Civit J, Magrí Aloy A, Schelde KM (2012) Inventory of manure processing activities in Europe

[CR14] Freundlich H (1907) Über die Adsorption in Lösungen vol 57U. 10.1515/zpch-1907-5723

[CR15] Gao N, Li Z, Quan C, Miskolczi N, Egedy A (2019). A new method combining hydrothermal carbonization and mechanical compression in-situ for sewage sludge dewatering: Bench-scale verification. J Anal Appl Pyrolysis.

[CR16] Gasco G, Paz-Ferreiro J, Alvarez ML, Saa A, Mendez A (2018). Biochars and hydrochars prepared by pyrolysis and hydrothermal carbonisation of pig manure. Waste Manag.

[CR17] Ghanim BM, Pandey DS, Kwapinski W, Leahy JJ (2016). Hydrothermal carbonisation of poultry litter: Effects of treatment temperature and residence time on yields and chemical properties of hydrochars. Bioresour Technol.

[CR18] Huang R, Tang Y (2015). Speciation dynamics of phosphorus during (hydro)thermal treatments of sewage sludge. Environ Sci Technol.

[CR19] Huang R, Tang Y (2016). Evolution of phosphorus complexation and mineralogy during (hydro)thermal treatments of activated and anaerobically digested sludge: insights from sequential extraction and P K-edge XANES. Water Res.

[CR20] Inoue S, Sawayama S, Ogi T, Yokoyama SY (1996). Organic composition of liquidized sewage sludge. Biomass Bioenergy.

[CR21] Kambo HS, Dutta A (2015). Comparative evaluation of torrefaction and hydrothermal carbonization of lignocellulosic biomass for the production of solid biofuel. Energy Convers Manag.

[CR22] Kang S, Li X, Fan J, Chang J (2012). Characterization of hydrochars produced by hydrothermal carbonization of lignin, cellulose, d-xylose, and wood meal. Ind Eng Chem Res.

[CR23] Kelessidis A, Stasinakis AS (2012). Comparative study of the methods used for treatment and final disposal of sewage sludge in European countries. Waste Manag.

[CR24] Langmuir I (1918) The adsorption of gases on plane surfaces of glass, mica and platinum. J Am Chem Soc 345. 10.1021/ja02242a004

[CR25] Latham KG, Jambu G, Joseph SD, Donne SW (2014). Nitrogen doping of hydrochars produced hydrothermal treatment of sucrose in H2O, H2SO4, and NaOH. ACS Sustain Chem Eng.

[CR26] Lu H, Zhang W, Yang Y, Huang X, Wang S, Qiu R (2012). Relative distribution of Pb2+ sorption mechanisms by sludge-derived biochar. Water Res.

[CR27] Mäkelä M, Yoshikawa K (2016). Simulating hydrothermal treatment of sludge within a pulp and paper mill. Appl Energy.

[CR28] Mäkelä M, Benavente V, Fullana A (2015). Hydrothermal carbonization of lignocellulosic biomass: Effect of process conditions on hydrochar properties. Appl Energy.

[CR29] Melo TM (2018). Plant and soil responses to hydrothermally converted sewage sludge (sewchar). Chemosphere.

[CR30] Méndez A, Fidalgo JM, Guerrero F, Gascó G (2009). Characterization and pyrolysis behaviour of different paper mill waste materials. J Anal Appl Pyrolysis.

[CR31] Muhammad Nasir I, Mohd Ghazi TI (2015). Pretreatment of lignocellulosic biomass from animal manure as a means of enhancing biogas production. Eng Life Sci.

[CR32] Ostman M, Fick J, Tysklind M (2018). Detailed mass flows and removal efficiencies for biocides and antibiotics in Swedish sewage treatment plants. Sci Total Environ.

[CR33] Rafatullah M, Sulaiman O, Hashim R, Ahmad A (2010). Adsorption of methylene blue on low-cost adsorbents: a review. J Hazard Mater.

[CR34] Rajkovich S, Enders A, Hanley K, Hyland C, Zimmerman AR, Lehmann J (2011). Corn growth and nitrogen nutrition after additions of biochars with varying properties to a temperate soil. Biol Fertil Soils.

[CR35] Raposo F, De La Rubia MA, Borja R (2009). Methylene blue number as useful indicator to evaluate the adsorptive capacity of granular activated carbon in batch mode: influence of adsorbate/adsorbent mass ratio and particle size. J Hazard Mater.

[CR36] Ro KS, Libra JA, Bae S, Berge ND, Flora JRV, Pecenka R (2018). Combustion behavior of animal-manure-based hydrochar and pyrochar. ACS Sustain Chem Eng.

[CR37] Rodriguez Correa C, Bernardo M, Ribeiro RPPL, Esteves IAAC, Kruse A (2017). Evaluation of hydrothermal carbonization as a preliminary step for the production of functional materials from biogas digestate. J Anal Appl Pyrolysis.

[CR38] Ronix A (2017). Hydrothermal carbonization of coffee husk: optimization of experimental parameters and adsorption of methylene blue dye. J Environ Chem Eng.

[CR39] Shen X, Huang G, Yang Z, Han L (2015). Compositional characteristics and energy potential of Chinese animal manure by type and as a whole. Appl Energy.

[CR40] Shi L, Zhang G, Wei D, Yan T, Xue X, Shi S, Wei Q (2014). Preparation and utilization of anaerobic granular sludge-based biochar for the adsorption of methylene blue from aqueous solutions. J Mol Liq.

[CR41] Smidt E, Parravicini V (2009). Effect of sewage sludge treatment and additional aerobic post-stabilization revealed by infrared spectroscopy and multivariate data analysis. Bioresour Technol.

[CR42] Standardization IOf (2017) ISO 21340:2017 - Test Methods for fibrous activated carbon

[CR43] Su Y, Zhu W, Gong M, Zhou H, Fan Y, Amuzu-Sefordzi B (2015). Interaction between sewage sludge components lignin (phenol) and proteins (alanine) in supercritical water gasification. Int J Hydrog Energy.

[CR44] Sun K, Jin J, Keiluweit M, Kleber M, Wang Z, Pan Z, Xing B (2012). Polar and aliphatic domains regulate sorption of phthalic acid esters (PAEs) to biochars. Bioresour Technol.

[CR45] Tasca AL, Puccini M, Gori R, Corsi I, Galletti AMR, Vitolo S (2019). Hydrothermal carbonization of sewage sludge: a critical analysis of process severity, hydrochar properties and environmental implications. Waste Manag.

[CR46] vom Eyser C, Palmu K, Schmidt TC, Tuerk J (2015). Pharmaceutical load in sewage sludge and biochar produced by hydrothermal carbonization. Sci Total Environ.

[CR47] vom Eyser C, Schmidt TC, Tuerk J (2016). Fate and behaviour of diclofenac during hydrothermal carbonization. Chemosphere.

[CR48] Wilk M, Magdziarz A, Jayaraman K, Szymańska-Chargot M, Gökalp I (2019). Hydrothermal carbonization characteristics of sewage sludge and lignocellulosic biomass. A comparative study. Biomass Bioenergy.

[CR49] Xia T, Huang H, Wu G, Sun E, Jin X, Tang W (2018). The characteristic changes of rice straw fibers in anaerobic digestion and its effect on rice straw-reinforced composites. Ind Crop Prod.

[CR50] Yahav Spitzer R, Mau V, Gross A (2018). Using hydrothermal carbonization for sustainable treatment and reuse of human excreta. J Clean Prod.

[CR51] Yang Y (2014). Biochar from Alternanthera philoxeroides could remove Pb(II) efficiently. Bioresour Technol.

[CR52] Yao Y, Gao B, Inyang M, Zimmerman AR, Cao X, Pullammanappallil P, Yang L (2011). Removal of phosphate from aqueous solution by biochar derived from anaerobically digested sugar beet tailings. J Hazard Mater.

[CR53] Zhang M, Gao B (2013). Removal of arsenic, methylene blue, and phosphate by biochar/AlOOH nanocomposite. Chem Eng J.

[CR54] Zhou S, Liang H, Han L, Huang G, Yang Z (2019). The influence of manure feedstock, slow pyrolysis, and hydrothermal temperature on manure thermochemical and combustion properties. Waste Manag.

