# Supplementary material for: The impact of hydrothermal carbonization on the surface functionalities of wet waste materials for water treatment applications
Source: Environ Sci Pollut Res Int. 2020 Apr 18;27(19):24369–79. doi: 10.1007/s11356-020-08591-w (PMC7326807; doi:10.1007/s11356-020-08591-w)
Supplement: Supplementary file 1 — (PDF 406 kb). [file 11356_2020_8591_MOESM1_ESM.pdf]

Supporting Information

Mirva Niinipuu, [†,‡] Kenneth G. Latham, [†] Jean-François Boily, [†] Magnus Bergknut, [§] Stina Jansson *,[†]

[†] Department of Chemistry, Umeå University, SE-90187, Umeå, Sweden

[‡] Industrial Doctoral School, Umeå University, SE-90187, Umeå, Sweden

[§] MTC-Miljötekniskt Center AB, Dåva Energiväg 8, SE-90595, Umeå, Sweden

* Corresponding author: stina.jansson@umu.se

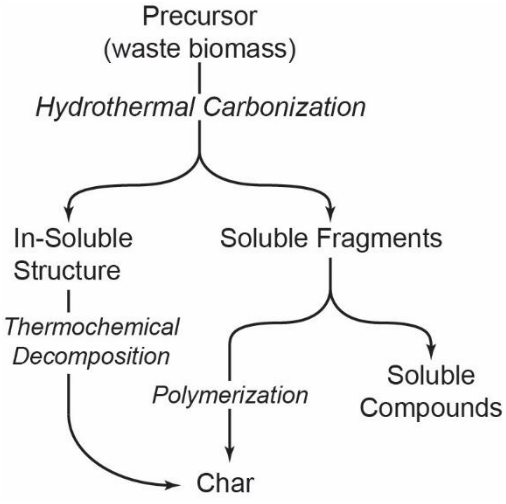

Figure S1. Overall reaction scheme for the HTC of waste biomass.

Table S1. Composition of the studied feedstocks

		Horse manure	Biosludge	Fiber sludge	Sewage sludge
C	%	42.3	37.6	37.8	28.7
S	%	0.16	1.27	0.12	0.99
N	%	1.00	5.64	0.25	3.26
H	%	6.38	6.46	6.04	5.48
Si	mg/kg DS	20500	23500	9470	31500
Al	mg/kg DS	4010	17300	2280	21800
Ca	mg/kg DS	4690	19500	54900	21000
Fe	mg/kg DS	3110	3780	1880	82900
K	mg/kg DS	9770	2600	913	3130
Mg	mg/kg DS	1800	3300	671	2550
Mn	mg/kg DS	155	8540	102	334
Na	mg/kg DS	3240	4530	583	1800
P	mg/kg DS	2110	10100	209	35100
Ti	mg/kg DS	185	323	132	1400
Loss on ignition	%	91.4	83	89	62.2
Ba	mg/kg DS	54.1	653	69.2	242
Be	mg/kg DS	<0.5	0.554	<0.5	0.85
Co	mg/kg DS	<3	<3	<3	7.87
Cr	mg/kg DS	<10	20.1	<10	36.5
Nb	mg/kg DS	<1	0.959	<1	2.35
Sc	mg/kg DS	<1	1.34	<1	2.23
Sr	mg/kg DS	30.3	60	49.7	110
V	mg/kg DS	4.19	8.43	2.55	23.3
W	mg/kg DS	<1	32.3	<1	3.32
Y	mg/kg DS	1.38	3.18	1.15	9.46
Zr	mg/kg DS	4.45	4.71	4.88	23.6

Table S2. Weight of the batch and dry material contents of the undiluted and diluted feedstocks and dry material based yield.

	Weight batch (g)	Dry material, raw (%)	Dry material after dilution (%)	Yield (%)
Horse manure 180	450.08	38%	19%	74%
Horse manure 220	465.86	38%	19%	66%
Horse manure 260	469.52	38%	19%	48%
Biosludge180	601.80	10%	10%	54%
Biosludge 220 *	608.07	10%	10%	47%
Biosludge 220-2 *	646.14	10%	10%	49%
Biosludge 260 **	635.39	10%	10%	44%
Biosludge 260-2 **	615.93	10%	10%	44%
Sewage sludge 180	595.73	30%	22%	83%
Sewage sludge 220	596.91	30%	22%	76%
Sewage sludge 260	600.13	30%	22%	70%
Fiber sludge 180	621.52	28%	19%	90%
Fiber sludge 220	611.96	28%	19%	70%
Fiber sludge 260	616.22	28%	18%	35%

* biochars from Biosludge 220 and 220-2 were pooled, ** biochars from Biosludge260 and 260-2 were pooled

Table S3. Positions and assignments of the main peaks displayed in the raw materials and those treated at 260℃.

	Sewage Sludge		Biosludge		Fiber Sludge		Horse Manure	
Assignment	Raw	260℃	Raw	260℃	Raw	260℃	Raw	260℃
-OH (kaolinite)	-	-	-	3695	-	-	-	-
-OH	3276	3236	3280		3325	3330	3323	3286
aliphatic C-H asym	2922	2923	2925	2925	2920	2925	2920	2923
aliphatic C-H sym	2851	2852	2850	2854	2851	2851	2850	2852
Non-Conjugated C=O	1731	-	1730	-	-	-	1731	-
Conjugated C=O	-	-	-	-	-	1695	-	1695
Amide I, OH deformation kaolinite	1637	-	1637	-	-	-	-	-
C=C Aromatic	-	-	-	1593	-	1596	1595	1596
Amine II	1537		1533					
Ar/Ar-OH	1514	-	1535	-	-	-	1510	1512
	-	1442	-	1438	-	-	-	1450
CH/COO^-	1419	-	1417	-	1417	1415	1419	-
$-CH_2-$	-	-	-	-	1375	-	1373	-
C-O on Guaiacol Ring	1259	-	-	-	-	1269	1259	1265
C-O on Syringyl Ring	-	-	1228	-	-	1209	-	1205
C-O-C	-	-	-	-	1203	-	-	-
C-O-C	-	-	-	-	1159	-	-	1118
C-O-C, C-OH	-	-	1031	1029	1028	1033	1024	1029
C-OH/Si-O-Si	1008	-	1008	1006	-	-	-	-
Si-O-Si	-	985	-	-	-	-	-	-
C-H out of plane	-	-	914	912	-	-	-	-
C-H out of plane	-	-	-	-	896	-	-	-
C-H out of plane	-	-	-	-	871	871	-	-
	-	858	-	-		-	-	-

Table S4. Binding energies (eV) of each carbon functionality and their proportion (%) in each sample.

Sample	C-C, C-H (eV / %)	C-OH (eV / %)	O-C-O, C=O (eV / %)	COOH (eV / %)
Sewage sludge 180	285.0/48.3	286.5/14.5	288.4/5.0	289.4/1.2
Sewage sludge 220	285.0/50.5	286.5/15.6	288.4/4.9	-
Sewage sludge 260	285.0/57.8	286.4/10.9	288.2/3.3	289.1/1.3
Biosludge 180	285.0/50.4	286.5/16.6	288.3/4.5	289.2/2.0
Biosludge 220	285.0/56.2	286.4/12.9	287.8/4.1	288.9/2.5
Biosludge 260	285.0/58.3	286.3/11.8	288.1/2.7	289.1/1.0
Fiber sludge 180	285.0/32.7	286.7/28.7	288.2/8.3	289.8/1.5
Fiber sludge 220	285.0/39.4	286.8/25.6	288.4/6.3	289.6/1.3
Fiber sludge 260	285.0/64.7	286.4/12.8	288.1/3.1	289.3/2.0
Horse manure 180	285.0/39.4	286.7/28.3	288.4/5.0	289.6/1.1
Horse manure 220	285.0/40.8	286.7/28.2	288.4/3.6	289.3/1.1
Horse manure 260	285.0/66.3	286.4/14.3	287.8/2.5	289.1/1.3

Table S5. Non-linear Freundlich and Langmuir model parameters

	Freundlich model			Langmuir model		
Sample	Kf	n	R2	Qmax	K_L	R2
Sewage sludge 220	15.124	4.244	0.9554	40.923	0.382	0.9847
Fiber sludge 220	12.437	3.728	0.9690	40.390	0.215	0.9779
Biosludge 180	18.956	3.430	0.9494	58.837	0.329	0.9949
Biosludge 220	19.902	2.911	0.9737	68.339	0.298	0.9928
Biosludge 260	10.790	3.430	0.9735	42.330	0.107	0.9609
Horse manure 180	7.611	3.192	0.9973	35.268	0.074	0.9506
Horse manure 220	5.829	3.060	0.9951	31.145	0.051	0.9445
Horse manure 260*	0.052	0.977	0.6158	117.646	0.001	0.6169

* Insufficient model fit
